# Morphological and Molecular Data Reveal Three Distinct Populations of Indian Wild Rice *Oryza rufipogon* Griff. Species Complex

**DOI:** 10.3389/fpls.2018.00123

**Published:** 2018-02-07

**Authors:** Balwant Singh, Nisha Singh, Shefali Mishra, Kabita Tripathi, Bikram P. Singh, Vandna Rai, Ashok K. Singh, Nagendra K. Singh

**Affiliations:** ^1^National Research Centre on Plant Biotechnology, New Delhi, India; ^2^Division of Genetics, Indian Agricultural Research Institute, New Delhi, India

**Keywords:** germplasm collection and conservation, *pSINE1* marker, genetic diversity, population structure, Indian wild rice

## Abstract

Wild relatives of crops possess adaptive mutations for agronomically important traits, which could play significant role in crop improvement for sustainable agriculture. However, global climate change and human activities pose serious threats to the natural habitats leading to erosion of genetic diversity of wild rice populations. The purpose of this study was to explore and characterize India’s huge untapped wild rice diversity in *Oryza rufipogon* Griff. species complex from a wide range of ecological niches. We made strategic expeditions around diversity hot spots in 64 districts of nine different agro-climatic zones of the country and collected 418 wild rice accessions. Significant variation was observed among the accessions for 46 morphological descriptors, allowing classification into *O. nivara, O. rufipogon*, and *O. sativa f. spontanea* morpho-taxonomic groups. Genome-specific *pSINE1* markers confirmed all the accessions having AA genome, which were further classified using ecotype-specific *pSINE1* markers into annual, perennial, intermediate, and an unknown type. Principal component analysis revealed continuous variation for the morphological traits in each ecotype group. Genetic diversity analysis based on multi-allelic SSR markers clustered these accessions into three major groups and analysis of molecular variance for nine agro-climatic zones showed that 68% of the genetic variation was inherent amongst individuals while only 11% of the variation separated the zones, though there was significant correlation between genetic and spatial distances of the accessions. Model based population structure analysis using genome wide bi-allelic SNP markers revealed three sub-populations designated ‘Pro-Indica,’ ‘Pro-Aus,’ and ‘Mid-Gangetic,’ which showed poor correspondence with the morpho*-*taxonomic classification or *pSINE1* ecotypes. There was Pan-India distribution of the ‘Pro-Indica’ and ‘Pro-Aus’ sub-populations across agro-climatic zones, indicating a more fundamental grouping based on the ancestry closely related to ‘Indica’ and ‘Aus’ groups of rice cultivars. The Pro-Indica population has substantial presence in the Eastern Himalayan Region and Lower Gangetic Plains, whereas ‘Pro-Aus’ sub-population was predominant in the Upper Gangetic Plains, Western Himalayan Region, Gujarat Plains and Hills, and Western Coastal Plains. In contrast ‘Mid-Gangetic’ population was largely concentrated in the Mid Gangetic Plains. The information presented here will be useful in the utilization of wild rice resources for varietal improvement.

## Introduction

Rice genetic diversity has laid the foundation of breeding programs around the world, however, breeders have exploited mostly landraces and the traditional rice varieties for this purpose with limited genetic advance. Wild rice species are rich source of genetic diversity and have genes for yield-enhancing traits, hybrid rice production, and tolerance to biotic and abiotic stresses, some of which have been introgressed into cultivated rice (Brar and Khush, 1997). Crop wild relatives are the key genetic resource for widening the genetic base of modern rice cultivars (Tanksley and McCouch, 1997). The broader genetic base of cultivars is essential to enhance crop productivity both by utilizing and enhancing crop yield potential as well as reducing the yield losses (Brar and Khush, 2003). Twenty-two wild rice species have been identified representing ten genomes, namely AA, BB, CC, EE, FF, GG, BBCC, CCDD, HHKK, and HHJJ (Lu et al., 2009). Two of these species, namely *O. rufipogon* Griff. and *O. nivara* Sharma et Shastry are of common occurrence in India (Sharma and Shastry, 1965), though *O. officinalis* and *O. granulata* have also been reported (Patra et al., 2008). However, internationally acclaimed ‘The Plant List’ database of all plant species^1^ lists *O. rufipogon* Griff. as the accepted species and *O. nivara* Sharma et Shastry as a synonym of this because these two easily cross-hybridize with each other in the natural populations. Hence, recent studies have grouped *O. nivara and O. rufipogon* together in a single *O. rufipogon* Griff. species complex (ORSC) (Kim et al., 2016). There have been landmark introgressions of agronomically useful genes from wild rice into cultivars, but such examples are rare, e.g., grassy stunt virus (Khush and Ling, 1974), bacterial leaf blight resistance gene *Xa-21* (Khush et al., 1990), blast resistance genes *Pi-9* (Amante-Bordeos et al., 1992), *Pi-40(t)* (Jeung et al., 2007), BPH resistance (Yan et al., 1997; Li et al., 2006; Zhang et al., 2014) and L-myo-inositol 1-phosphate synthase gene for salt tolerance (Das-Chatterjee et al., 2006). The cross-incompatibility between species is a major hindrance to their effective utilization in rice improvement (Brar and Khush, 2003). However, six of the 22 wild *Oryza* species belonging to the AA genome are compatible with the cultivated rice and could play an important role in enhancing rice productivity (Chang, 1976).

The changing global climate, increasing human population, developmental pressure, and other human activities pose serious threat to natural habitats and consequently to the genetic diversity of the wild rice populations (Vaughan and Chang, 1992). Therefore, to conserve representative wild rice germplasm, strategic expeditions are required around all the agro-climatic regions of the world, threatened zones, areas having genetically isolated smaller populations and diversity hot spots (Vaughan and Chang, 1992). Initiatives for collection of wild rice have been taken in India and abroad with the purpose of conservation and utilization of this highly valued germplasm (Ng et al., 1983; Second and Ghesquière, 1985; Rana and Sharma, 1990; Vaughan, 1994; Rai, 1999; Patra et al., 2008). International Rice Genebank Collection Information System (IRGCIS) of IRRI showed total 4,610 accessions of wild rice species, 838 of which were of Indian origin^2^ (as accessed on 02 February 2016). Indian Genebank at the National Bureau of Plant Genetic Resources (NBPGR) listed 354 accessions of *O. rufipogon* and 723 accessions of *O. nivara*^3^ (as accessed on 02 February 2016).

The germplasm utilization strategies such as establishment of a core set which efficiently captures a high percentage of the genetic variation are dependent on genetic diversity and population structure analysis (Mackay, 1995; Upadhyaya, 2015). Efficient techniques such as focused identification of germplasm strategies (FIGS) for the identification of specific adaptive traits in a germplasm collection further speed up the breeding process (Mackay and Street, 2004). Characterization, classification, and evaluation of genetic structure of the newly acquired germplasm help in effective utilization strategies (Vaughan et al., 2005). Morphological traits have long been used for the taxonomic classification and variation studies in wild rice, but they provide a limited basis of understanding the species variation (Vaughan, 1994). Further, cytogenetics, molecular markers, and genomic tools have been employed for identification and differentiation of ecologically and genetically distinct species and ecotypes (Aggarwal et al., 1997). Genome-specific repetitive DNA elements have been used for analyzing relationship between different genomes of rice (Cordesse et al., 1992; McIntyre and Winberg, 1998). Isozyme markers were used first for genetic diversity and population structure analysis in rice (Glaszmann, 1987), however, recent advancements in genomics offer excellent tools to conduct unbiased genetic diversity, population structure and phylogenetic studies. Molecular markers such as SSR (Yibo et al., 2010; Singh N. et al., 2013), *pSINE1* (Ohtsubo et al., 2004) and SNP (Xu et al., 2012) have been used for classification, genetic diversity, and population structure analysis of *Oryza* species. Short interspersed nuclear elements (SINE) are found in almost all eukaryotes and are excellent candidates for evolutionary analysis due to their specific characters. Plant SINE1 (*pSINE1*) retrotransposons were first identified in *O. sativa* and *O. glaberrima* by Umeda et al. (1991). Different families of *pSINE1* elements, each having large numbers of *pSINE1* markers were identified among *O. sativa* and *O. officinalis* complexes (Hirano et al., 1994; Motohashi et al., 1997; Cheng et al., 2002, 2003; Xu et al., 2007). Complementing morphological descriptors with appropriate molecular techniques is an effective method for genetic diversity and species characterization. Even though Indian subcontinent is known to have at least six different wild *Oryza* species, *O. nivara* and *O. rufipogon* are the most abundant and highly adapted to the rice growing environments and hence likely source of useful new genes for rice varietal improvement. Morphological descriptors have been used in the past for taxonomic classification of *Oryza* species, but these are based on expression of limited number of genes, which is further modulated by environmental conditions. Hence, there is need to use robust molecular markers for this purpose. The *pSINE1* elements are highly reproducible markers for determining the genomic organization and species of wild rice (Cheng et al., 2003; Xu et al., 2007). Multi-allelic SSR markers are more efficient than SNP markers for genetic diversity analysis because resolving power for clustering increases with the number of alleles per locus. On the other hand highly reproducible bi-allelic SNP markers are better suited for population structure analysis (Coates et al., 2009; Singh N. et al., 2013). Therefore, we used *pSINE1* markers for species and ecotype identification, SSR markers for genetic diversity analysis and a newly designed 48-plex SNP array for population structure analysis in a set of 418 ORSC wild rice accessions, most of which were newly collected through exploration expeditions to different agro-climatic zones of India.

## Materials and Methods

### Collection of Wild Rice Accessions

In order to conserve and utilize the fast-depleting Indian wild rice resource, different agro-climatic zones categorized by the Planning Commission of India (Khanna, 1989) were explored and a total of 418 accessions were collected and characterized during 2010–2014. For each sample, two to three hundred mature seeds were collected from the panicles of multiple wild rice plants because of uneven maturity and shattering problems. Complete passport data including GPS information, habitat information, photographs of the seeds, plants and collection site and interviews of the local farmers were recorded and are stored in a web-enabled database^4^. The 418 accessions include 360 freshly collected accessions, designated NKSWR001–NKSWR396, and 58 accessions obtained from NBPGR, New Delhi (**Supplementary Table S1**). Minimum sampling distance between accessions was kept 20 km. Earlier Kiambi et al. (2005) sampled wild rice accessions from 15 km apart considering climatic and demographic variations. We also analyzed five accessions each of Aus (Apo, FR13A, Dihawa, Nagina 22, and Rajbhog) and Indica (CR 1009, IR 64, Ranjit, Samba Mahsuri, and Swarna) rice cultivars used as reference for the classification of wild rice accessions based on their similarity to the rice cultivar groups.

### Morphological and Taxonomic Characterization

The collected wild rice accessions were planted in *Kharif* season of each year from 2011 to 2014 at Indian Agricultural Research Institute, New Delhi, India. Each accession was planted in a single row with 10 plants per row and grid spacing of 50 cm by 50 cm. Each accession was evaluated once in a single year and observations were recorded on three random plants for 46 morphological descriptors (**Supplementary Table S2**). Traits were evaluated visually and a standard method was followed for transforming each trait value into numeric form using descriptors for wild and cultivated rice (Bioversity International et al., 2007). Morphological traits including growth habit, i.e., culm angle, panicle type, anther length, awning, seed shattering and seed morphology were used for taxonomic differentiation of *O. rufipogon, O. nivara*, and *O. sativa f. spontanea* according to published criteria (Sharma and Shastry, 1965; Vaughan, 1994; Suh et al., 1997). *O. nivara* was identified with semi-spreading growth habit, close or semi-compact panicles, less than 3 mm long anthers, long thick awns, low to high seed shattering and bold seeds with length to breadth ratio of less than three. *O. rufipogon* was identified by spreading growth habit with culm angle of close to 90°, open panicles, anther length larger than 3.5 mm, long thin awns, moderate to high seed shattering and relatively slender seeds with length to breadth ratio of three or above (Vaughan, 1994). *O. sativa f. spontanea* was identified with erect pant habit, close or semi-compact panicles, less than 3 mm long anthers, shorter than 6 mm awns, low to moderate seed shattering, and bold seeds with length to breadth ratio of less than three (Suh et al., 1997).

### Genomic DNA Extraction and Analysis of *pSINE1* and HvSSR Markers

For molecular characterization, DNA was extracted from the leaves of a single representative plant grown from the original seeds collected during the field expedition using the method of Murray and Thompson (1980) with minor modifications. The leaf tissue ground in liquid N_2_ was incubated in DNA extraction buffer (800 μl) at 65°C for 45 min in a water bath, mixed with equal volume of phenol:chloroform:isoamyl alcohol (25:24:1) and centrifuged at 10,000 rpm for 15 min. The aqueous phase was transferred to a fresh tube and DNA was precipitated overnight with equal volume of chilled isopropanol, dissolved in 100 μl of TE and quantified by agarose gel electrophoresis along with known amounts of bacteriophage λ DNA and also by NanoDrop 8000 spectrophotometer (Thermo Scientific). For species and ecotype identification we used nine different *pSINE1* markers according to Cheng et al. (2003). Twenty-four genome wide HvSSR markers (Singh H. et al., 2010), one from each of the chromosome arms of rice were used for the genetic diversity analysis. PCR amplification was carried out in 10 μl reaction volume in a Bio-Rad thermal cycler, using 20 ng of genomic DNA, 20 picomole of each primer, 1.0 μl of 10× PCR buffer containing 17.5 mM MgCl_2_, 0.15 μl of 2.5 mM dNTP mixture, 0.12 μl (0.6 unit) of Taq DNA polymerase (Vivantis), and 5.73 μl of H_2_O. The temperature profile of the PCR amplification was initial denaturation at 95°C for 5 min, followed by 35 cycles of denaturation at 95°C for 45 s, annealing at 50–56°C for 40 s, extension at 72°C for 1 min, and a final extension at 72°C for 10 min. PCR products were separated by electrophoresis in 2.5% Metaphor agarose gel for *pSINE1* markers and 4% Metaphor agarose gel for SSR markers.

### 48-plex GoldenGate SNP Assay and Population Structure Analysis

A genome-wide 48-plex SNP assay was designed using four SNPs for each of the 12 rice chromosomes, located at nearly equidistant positions (**Supplementary Figure S1**). To design the new assay a 36-plex Sequenom MassARRAY SNP assay described earlier (Singh A. et al., 2010; Singh N. et al., 2013) was upgraded by adding one more SNP on each chromosome to fill the gaps and make them equidistant. SNPs were identified *in silico* from the OryzaSNP database^5^, taking 60 bp on either side of the SNP position. For designing the assay, sequence-type preliminary input assay file was designed and submitted to the assay design tool (ADT) of Illumina. After evaluation, the ADT-generated output SNP score file was sent for Oligo Pool Assay (OPA) synthesis. To customize the array, VeraCode technology was employed. The final output score and design ability rank signifies the quality of SNPs and success of OPA. The output score value was kept in the range of 0.600–1.1 and the design ability rank was 1.0 (**Supplementary Table S3**). SNP genotyping was performed according to the protocol provided by Illumina for VeraCode GoldenGate Genotyping Assay (Catalog#VC-901-1001). Data analysis was performed with Genome Studio v2011.1 software with an initial Gentrain threshold of 0.25. Plink software was used to extract the data from genome studio for population structure analysis using STRUCTURE v2.3.4 software (Pritchard et al., 2000). Previous studies have shown that *Oryza nivara* Sharma et Shastry and *Oryza rufipogon* Griff. belong to a single species complex; therefore we analyzed their population structure together (Huang P.U. et al., 2012; Huang X. et al., 2012; Kim et al., 2016). To identify the population structure, a series of tests were performed to determine the optimal *K*-value, using a range of population clusters (*K* = 2–10, each with five independent runs/iterations) with both burn-in and replication values set at 50,000. The optimal *K*-value indicates the number of genetically distinct sub-populations while change in the log probability of the data between successive *K*-values signifies the Δ*K* (Evanno et al., 2005). Evanno parameters were calculated by online software Structure Harvester v 6.0 (Earl, 2012). Out of the 418 wild rice accessions analyzed in the present study 86 have been clustered earlier using a 50K SNP chip into three groups, two of which corresponded to ‘Indica’ and ‘Aus’ cultivar groups (Singh N. et al., 2015), we used these as reference for assigning ‘Pro-Indica’ and ‘Pro-Aus’ designations to the wild rice accessions. We also included in the present study five varieties each of ‘Indica’ and ‘Aus’ rice cultivars for re-verification of this classification.

### Statistical Analysis

Data for quantitative traits were evaluated in three replications and ANOVA was performed using SPSS Statistics v17 (SPSS Inc.). In order to evaluate correspondence between *pSINE1* markers and morphological traits, PCA analysis was performed using software STAR version 2.0.1 (2014). The clusters generated by *pSINE1* markers were used as one component while morphological traits were used as the second component. Dendrogram and statistical analysis of SSR markers was done by PowerMarker v3.25 (Lui and Muse, 2005) using CS Chord1967 genetic distance method (Cavalli-Sforza and Edwards, 1967) and dendrogram was constructed by the Unweighted Pair-Group Method of Arithmetic average (UPGMA). Bootstrap values placed at the nodes of the clusters were estimated using ‘FreeTree’ for SSR markers (Hampl et al., 2001) and ‘Phangorn’ package of ‘R software’ for SNP markers (Schliep, 2017). The analysis of molecular variance (AMOVA) was carried out to dissect the distribution of genetic diversity within and among species and sub-populations and the number of migrants per generation (Nm) was calculated using GenAlEx v6.5 software (Peakall and Smouse, 2012). The Mantel test was conducted to examine the isolation-by-distance pattern by analyzing correlation between genetic distance of wild rice populations and spatial distance of their geographical localities using the GenAlEx v6.5 software.

## Results

### Distribution and Abundance of ORSC Wild Rice in Different Regions of India

**FIGURE 1:**
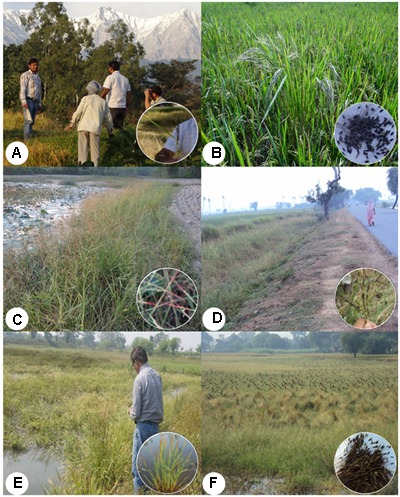
Typical ecological habitats at the collection sites of the Indian *Oryza rufipogon* Griff. species complex (ORSC) wild rice germplasm (insets showing panicles and seeds). **(A)** Western Himalayan Region in Himachal Pradesh, **(B)** Inside cultivated rice field in Uttarakhand, **(C)** Along sodic soils in Uttar Pradesh, **(D)** Along roadside strip in Bihar, **(E)** Road side shallow water lowland in Bihar, **(F)** Wild rice panicles tied by humans to prevent seed loss by shattering in Uttar Pradesh (written informed consent has been obtained from the subjects for the publication of their identifiable image).

Multiple expeditions were made for the collection of *O. nivara/O. rufipogon* and intermediate wild rice accessions belonging to the ORSC complex from 64 districts of 12 states representing nine agro-climatic zones of India (**Supplementary Table S1**). Keeping in view the typical wild rice habitats, collections were made from different ecological niches, including uncultivated land near rice fields, rice fields with poor weed control, along roadsides and canals, fresh water ponds, shallow and marshy lands, upland rice fields and nearby fallow lands, costal saline and other stress prone areas (**Figures 1A–F**). This covered a wide range of ecological niches for both annual (*O. nivara*) and perennial (*O. rufipogon*) ORSC wild rice populations. To minimize duplication and capture of representative diversity, wild rice seed samples were collected from sites minimum 20 km apart in remote villages, roadsides and undisturbed areas. To obtain full passport data, farmers, knowledgeable local persons and state university rice breeders were interviewed. The new accessions were collected from wide eco-geographical regions ranging from 14°N to 32°N latitude and 70°E to 93°E longitude and assigned accession numbers NKSWR001–NKSWR396 (**Supplementary Table S1**). Further, to find new sources of resistance to different biotic and abiotic stresses efforts were made to collect seeds of healthy looking plants growing in the stress-prone ‘hot spot’ areas. Accessions collected during 5 years of exploration were evaluated for different biotic and abiotic stresses, which further helped us revisit some of the areas with useful sources of diversity.

**FIGURE 2:**
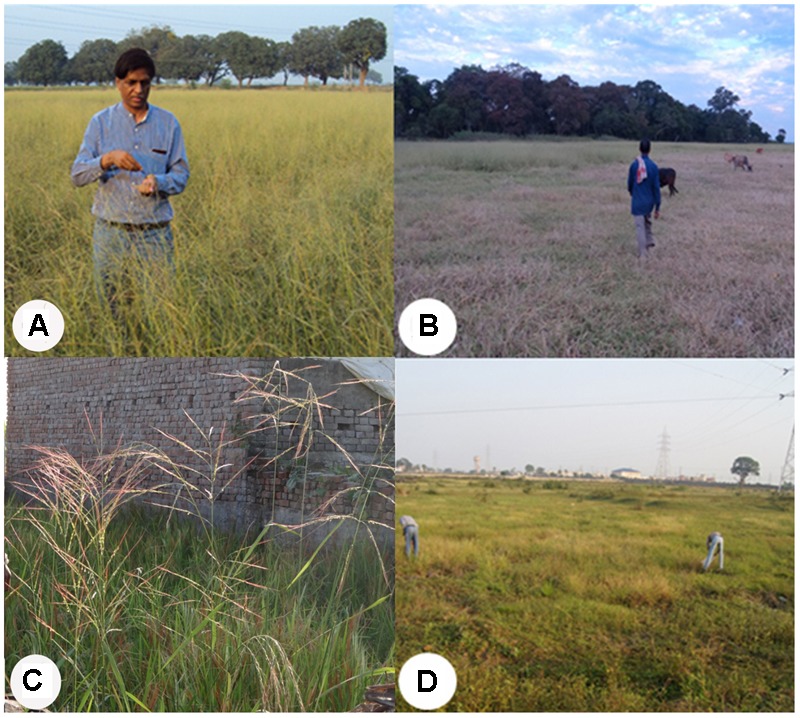
Collection sites showing large stands and threatened ecological habitats of the Indian ORSC wild rice. **(A)** Large shallow pond (*chhotaka pokhara*) full of wild rice in Gaya, Bihar. **(B)** Large wild rice stand on lowland river bank in Sibsagar, Assam. **(C)** Threatened by building construction in small town, Khalilabad, Uttar Pradesh. **(D)** Threatened by upcoming industrial complex in a metropolitan city Raipur, Chhattisgarh (written informed consent has been obtained from the subjects for the publication of their identifiable image).

From the 64 districts explored, we found that Sibsagar in Assam; Gaya and Patna in Bihar; Daboi in Gujarat; and Azamgarh, Ballia, Basti, Chandauli, and Siddharthnagar in Uttar Pradesh have plentiful pure stands of wild rice in large ponds and shallow lowland habitats (**Figures 1E,F, 2A,B**). Further, in these areas wild rice was plentiful on the roadsides in the protected buffer zone between the road and the farmers’ field. In the upland habitats of Chhattisgarh, Goa, Himachal Pradesh, and Uttarakhand, wild rice was often found growing in the middle of the paddy fields with poor weed management (**Figure 1B**). In general ORSC wild rice was found in the same habitats that have abundant rice cultivation and rarely in the areas devoid of rice farming. Large habitat with more than a hectare of pure stand of wild rice was rare throughout the Indian countryside. The shrinking habitat of wild rice is primarily due to clean cultivation with good weed management and developmental pressure from growing rural and small town population, loss of common uncultivated village land, rampant urbanization and industrialization. Quite often, wild rice was seen growing in the middle of the town within protected boundary walls on land waiting for building construction, which will surely disappear as the construction work is completed, e.g., Khalilabad in Uttar Pradesh and Raipur in Chhattisgarh (**Figures 2C,D**).

### Variation in Growth Habit and Morphological Traits of the Indian ORSC Wild Rice

**FIGURE 3:**
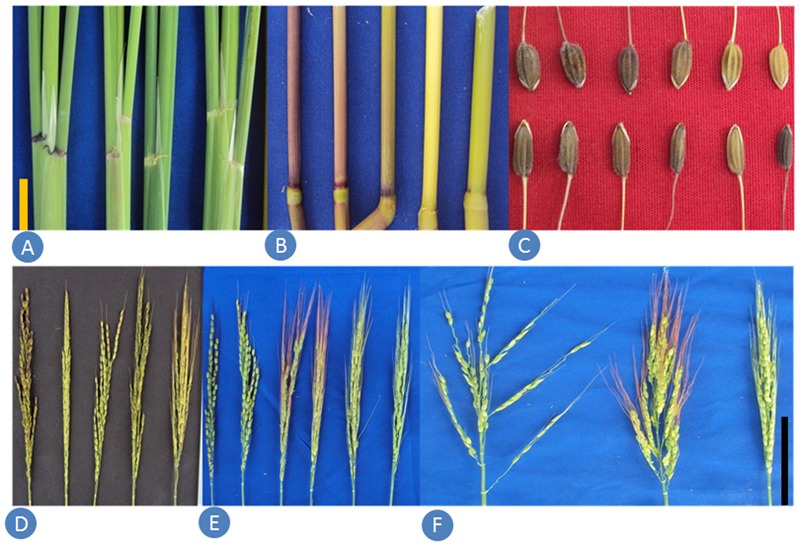
Samples of morphological variability in Indian ORSC wild rice accessions. **(A)** Auricle color, **(B)** internode pigmentation, **(C)** seed size and color, **(D)** extent of awning, **(E)** awn color and **(F)** panicle type.

The wild rice samples collected from remote villages were planted in the experimental fields at New Delhi and data were recorded for 46 phenotypic traits (descriptors) on all the 418 accessions (**Supplementary Table S2**). The analysis of variance for quantitative morphological traits revealed that mean sums of square values were highly significant and hence used for further analysis (**Supplementary Table S2**). A wide range of variability was observed for the qualitative morphological traits (**Figure 3**). Similarly, tremendous variability was measured for plant height, culm number, days to 50% flowering, grain length, grain width, panicle exertions, awning, awn color, awn length and anther length. Leaf blade color was green in 176 accessions, dark green in 163, purple margins in 49, purple tips in 17, light green in 11, and purple in one accession. Basal leaf sheath color was purple in 183 accessions, green in 152, light purple in 60, and purple lines in 21 accessions. Leaf angle was erect type in 244 accessions, droopy type in 130 accessions and horizontal type in 21 accessions. As for the growth habit, 115 accessions have spreading habit, 14 accessions have prostrate habit with small rhizomes and 89 have erect type with culm angle of lower than 60°. Panicles were close type in 64 accessions, open type in 119 and intermediate type in 234 accessions. High variability was also recorded for awning and awn color. Six classes of awning was recorded, namely no awns in 19 accessions, short and partly awned in 36, short and fully awned in 78, long and partly awned in four, and long and fully awned in 280 accessions. Six different awn colors were recorded, namely red in 152 accessions, straw in 127, purple in 32, black in 9, golden in 8 and white in the remaining 90 accessions. Panicle exertion also showed six classes, namely enclosed in three accessions, just exerted in 136, well exerted in 127, partly enclosed in 108, moderately well exerted in 21 and partly exerted in 22 accessions. Anther length varied from 1.6 mm in NKSWR009, NKSWR038, and NKSWR107 to 6.0 mm in NKSWR393. Panicle length ranged from 9.3 cm in NKSWR130 to 34.0 cm in NKSWR243. Plant height ranged from 37 cm in NKSWR096 to 226 cm in NKSWR391. Days to 50% flowering ranged from 77 days in NKSWR008 to 158 days in *O. nivara* 336698, 330630, and 330650. Based on the diagnostic morphological descriptors described in the methods section, 307 accessions were classified as *O. nivara* type, 66 accessions as *O. rufipogon* type and 45 accessions as intermediate (*O. sativa f. spontanea*) type (**Supplementary Table S2**).

### Classification of the Indian ORSC Wild Rice Accessions Based on *pSINE1* Markers

For an objective classification of the ORSC wild rice accessions in our collection, we analyzed nine different *pSINE1* markers on the 418 accessions and raw data are presented in **Supplementary Table S4**. Of these, six *pSINE1* markers (r1, r502, r601, r801, r904, and r705) are diagnostic markers for the classification of AA genome wild rice species (Cheng et al., 2003). Presence of marker r1 showing a PCR product of 740 bp is common to all the five AA genome species, while presence of r502 with PCR product of 380 bp is specific to ORSC (*O. rufipogon*) accessions and differentiates them from the other AA genome species. Here, *O. nivara* Sharma et Shastry is not considered a separate species but taken as an annual form of *O. rufipogon* Griff. Presence of marker r601 is specific to *O. barthii* whereas presence of both r601 and r801 is specific to *O. glumaepatula.* The remaining two AA genome species, viz. *O. meridionalis* and *O. longistaminata*, are confirmed by the presence of markers r904 and r705, respectively. We found that all the 418 accessions were positive for the *pSINE1*-r1 marker allele of 740 bp, indicating that each belonged to the AA genome. Furthermore, the ORSC-specific *pSINE1*-r502 marker allele of 380 bp was also present in all the 418 accessions, indicating that all belonged to the ORSC complex, namely *O. nivara, O. rufipogon* or their intermediate type. Marker *pSINE1*-r2 has been described to differentiate *O. rufipogon* from *O. nivara* (Yamanaka et al., 2003). Here, we found that only 23 of the 418 accessions with *pSINE1-*r2 marker allele of 421 bp could be classified as *O. rufipogon*, the remaining 395 were classified as *O. nivara.* Two *pSINE1* markers, namely *pSINE1-*r503 and *pSINE1-*r215 have been used to sub-group the ORSC accessions in to three different ecotypes (Xu et al., 2007). Thus, annual ORSC populations have presence of the markers alleles 500 bp and 900 bp, respectively; perennial populations have presence of only *pSINE1-*r503 allele of 500 bp while intermediate types have absence of both these marker alleles. Based on the analysis of these two *pSINE1* markers on 418 ORSC accessions, 117 were classified as annual (*O. nivara*), 132 as perennial (*O. rufipogon*), and 102 as intermediate type. Interestingly, the remaining 67 accessions were classified as unknown type because they showed a novel PCR amplicon size not described by Xu et al. (2007). Principal component analysis of the morphological traits and *pSINE1* ecotypes showed first two principal components accounting for 23.77% of the total variation. PC1 explained 13.62% of the total variance and it separated annual wild rice accessions from perennial and intermediate accessions, whereas PC2 explained 10.14% of the phenotypic variance but there was no clear grouping of the accessions (**Supplementary Figure S2**).

### Genetic Diversity and Geographical Isolation of Indian ORSC Wild Rice Populations

**FIGURE 4:**
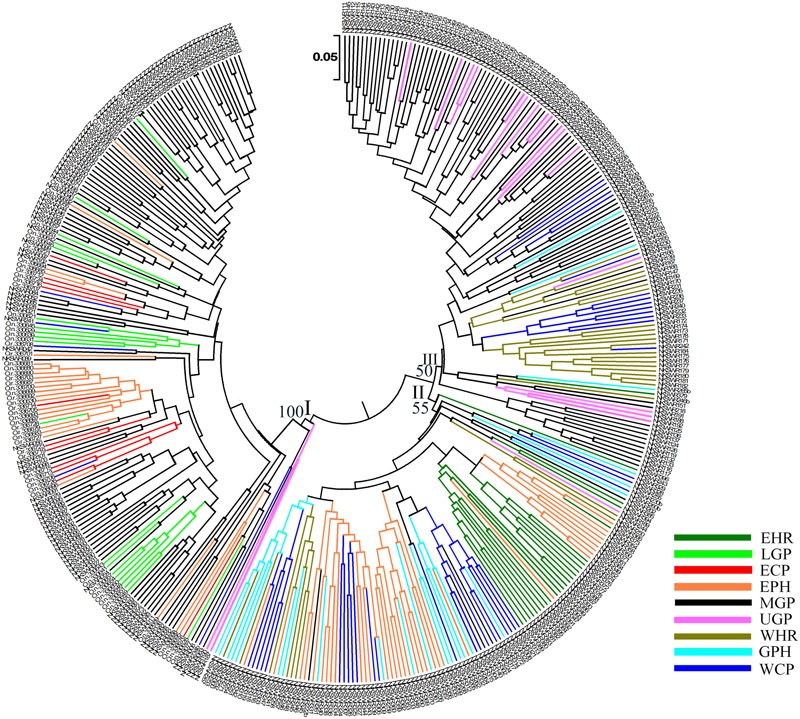
Unweighted pair-group method of arithmetic average based dendrogram of 418 Indian ORSC wild rice accessions collected form nine agro-climatic zones of India based on 24 genome-wide SSR markers. Agro-climatic zones are color-coded. EHR, Eastern Himalayan Region; LGP, Lower Gangetic Plains; ECP, Eastern Coastal Plains; EPH, Eastern Plateau and Hills; MGP, Mid Gangetic Plains; UGP, Upper Gangetic Plains; WHR, Western Himalayan Region; GPH, Gujarat Plains and Hills; WCP, Western Coastal Plains and Ghats.

To identify genetic diversity and interrelationship of individuals in our wild rice collection a set of 24 hyper variable SSR (HvSSR) markers, one from each arm of the 12 rice chromosomes was analyzed (**Supplementary Table S5**). The 24 SSR markers showed total 96 alleles, ranging from two alleles for HvSSR03-23 to six alleles for HvSSR01-22 locus. Major allele frequency (A) ranged from 0.33 (HvSSR05-37) to 0.90 (HvSSR06-34), gene diversity (G) from 0.17 (HvSSR06-34) to 0.75 (HvSSR07-33), heterozygosity (Ho) from 0.0 (HvSSR05-07) to 0.74 (HvSSR07-33), and polymorphic information content (PIC) from 0.16 (HvSSR06-34) to 0.72 (HvSSR07-33). Robustness and competency of SSR markers for studying overall molecular genetic diversity in rice germplasm has been reported earlier (Singh A. et al., 2013). We generated a dendrogram based on Chord frequency distance by UPGMA method, which grouped the accessions collected from nine agro-climatic zones into three major clusters *viz*. cluster-I, II, and III (**Figure 4**). Cluster analysis and distance metrics illustrated that no two accessions were identical, and the collection was genetically quite diverse. In general accessions collected from the same agro-climatic zone grouped together showing close genetic relationship as seen in the color-coded dendrogram (**Figure 4**). Among the cluster-specific agro-climatic populations, accessions of the EHR zone from Assam (dark green) were confined to cluster II, and those from LGP (light green) and ECP (red) zones were confined to cluster I. On the other hand MGP (black) and WCP (blue) accessions were present in all the three clusters, whereas EPH (orange), WHR (medium green), UGP (magenta), and GPH (cyan) accessions were present in two clusters each. This shows that at least six agro-climatic zones have ORSC populations belonging to more than one genetic lineage, with MGP and WCP wild rice populations being the most diverse.

Genetic characterization of four *pSINE1*-based ecotypes revealed that the mean observed (Ho) and expected (He) heterozygosity ranged from 0.102 (perennial) to 0.169 (intermediate) and from 0.585 (intermediate) to 0.637 (annual), respectively. The number of alleles (Na), effective alleles (Ne), and fixation index (F) were quite similar for all the four ecotypes (**Table 1**). Genetic characteristics of wild rice populations collected from nine agro-climatic zones of India showed wide range of observed (Ho) and expected (He) heterozygosity ranging from 0.065 for LGP to 0.247 for EHR and from 0.461 for ECP to 0.619 for MGP, respectively (**Table 2**). There was significant variation in Na values, ranging from 2.833 for ECP to 4.542 for MGP populations and *F*-values ranging from 0.549 for EHR to 0.888 for LGP population. Analysis of molecular variance (AMOVA) generated from the genetic distance matrix showed significant variations between populations of different ecotypes and different agro-climatic zones, among individuals and within individuals. Within individual variation is accessed by the GenAlEx software due to varying levels of heterozygosity of marker loci from different segments of the genome. This statistics is not available for pure homozygous individuals. AMOVA for *pSINE1* marker-based ecotypes showed the largest proportion of genetic variation (73.6%) was among individuals, followed by 21.4% within individuals and only a small proportion (5%) was due to differences among ecotypes (**Supplementary Table S6**). To partition the molecular variation on the basis of geographical origin, the nine populations from different agro-climatic zones were analyzed, which revealed a comparatively higher variation of 11% among populations, but the largest proportion of variation (68%) was still among individuals followed by 21% within individuals (**Table 3**). The Mantel test showed low but significant correlation (*r*^2^ = 0.073, *P* < 0.001) between the genetic distance and geographic distance (UTM) matrix of populations from different agro-climatic zones. To measure the level of gene flow between wild rice populations from different agro-climatic zones, a matrix was generated for the number of migrants per generation (Nm) and Fst between each pair of all agro-climatic zones. Overall average Fst was 0.108 at *P* < 0.001 and average Nm was 1.786 for all the agro-climatic zones. Pairwise Nm analysis based on Fst data matrix showed a high level of gene flow with higher Nm values between geographically adjacent populations as compared to distant populations (**Table 4**). For example the highest Nm value of 5.234 was between UGP and WHR followed by 4.473 between WCP and GPH regions, whereas the lowest Nm value was 0.571 between EHR and LGP followed by 0.700 between GPH and ECP regions.

**Table 1:** Genetic characteristic of four *pSINE1* characterized ecotypes of Indian *Oryza rufipogon* species complex wild rice accessions based on HvSSR markers.

Ecotype		Ns	Na	Ne	I	Ho	He	uHe	F
A	Mean	117	4.750	3.008	1.190	0.149	0.634	0.637	0.789
	SE		0.257	0.194	0.060	0.035	0.024	0.024	0.045
I	Mean	102	4.583	2.700	1.103	0.169	0.582	0.585	0.765
	SE		0.294	0.204	0.067	0.049	0.029	0.029	0.063
P	Mean	132	4.500	2.936	1.179	0.102	0.625	0.627	0.855
	SE		0.255	0.173	0.058	0.030	0.027	0.027	0.039
U	Mean	67	4.708	2.975	1.198	0.160	0.637	0.641	0.768
	SE		0.266	0.177	0.055	0.032	0.022	0.022	0.042

*A, annual; I, intermediates; P, perennial; U, unknown; Ns, sample size; Na, number of alleles; Ne, number of effective alleles; I, information index; Ho, observed heterozygosity; He and uHe, expected and unbiased expected heterozygosity; F, fixation index.*

**Table 2:** Genetic characteristics of 418 Indian *Oryza rufipogon* species complex wild rice accessions from nine different agro-climatic zones based on HvSSR markers.

Region		Ns	Na	Ne	I	Ho	He	uHe	F
Eastern Coastal Plains	Mean	11	2.833	2.150	0.790	0.072	0.461	0.483	0.875
(ECP)	SE		0.223	0.176	0.083	0.033	0.044	0.046	0.054
Eastern Himalayan Region	Mean	25	3.625	2.281	0.888	0.247	0.486	0.496	0.549
(EHR)	SE		0.239	0.198	0.080	0.047	0.04	0.042	0.079
Eastern Plateau and Hills	Mean	60	4.250	2.606	1.057	0.108	0.579	0.584	0.830
(EPH)	SE		0.277	0.163	0.064	0.029	0.027	0.028	0.040
Gujarat Plains and Hills	Mean	21	3.333	2.319	0.880	0.179	0.506	0.518	0.708
(GPH)	SE		0.299	0.180	0.082	0.047	0.040	0.041	0.072
Lower Gangetic Plains	Mean	20	3.167	2.243	0.881	0.065	0.512	0.525	0.888
(LGP)	SE		0.187	0.132	0.063	0.026	0.033	0.033	0.044
Middle Gangetic Plains	Mean	187	4.542	2.942	1.165	0.122	0.619	0.60	0.830
(MGP)	SE		0.262	0.191	0.065	0.037	0.031	0.031	0.048
Upper Gangetic Plains	Mean	18	3.833	2.580	1.026	0.238	0.563	0.579	0.638
(UGP)	SE		0.267	0.182	0.071	0.065	0.033	0.034	0.093
Western Coastal Plains and	Mean	44	4.417	2.856	1.127	0.155	0.598	0.605	0.781
Ghats (WCP)	SE		0.240	0.200	0.071	0.037	0.035	0.036	0.049
Western Himalayan Regions	Mean	32	4.125	2.704	1.077	0.195	0.584	0.593	0.716
(WHR)	SE		0.284	0.189	0.067	0.054	0.030	0.031	0.073
Total	Mean	418	3.792	2.520	0.988	0.153	0.545	0.556	0.756
	SE		0.092	0.062	0.025	0.015	0.012	0.012	0.022

*N, sample size; Na, number of alleles; Ne, number of effective alleles; I, information index; Ho, observed heterozygosity; He, expected heterozygosity; uHE, unbiased expected heterozygosity; F, fixation index.*

**Table 3:** AMOVA summary of Indian *Oryza rufipogon* species complex wild rice populations from nine different agro-climatic zones of India based on HvSSR markers.

Source	Df	SS	MS	Est. var.	Percent variation	*P*-value
Among populations	8	640.543	80.068	0.860	11	<0.001
Among individuals	409	5143.091	12.575	5.444	68	<0.001
Within individuals	418	705.000	1.687	1.687	21	<0.001
Total		6,488.634		7.990	100	

*SS, total sum of squares; MS, mean sum of square.*

**Table 4:** Fst-based pairwise migrants per generation (Nm) among Indian *Oryza rufipogon* species complex wild rice populations from nine different agro-climatic zones of India.

	ECP	EHR	EPH	GPH	LGP	MGP	UGP	WCP
EHR	0.709						
EPH	2.464	2.097					
GPH	0.700	2.292	2.965				
LGP	3.221	0.571	1.339	0.609			
MGP	3.320	1.234	3.221	1.876	1.941		
UGP	1.016	1.038	1.657	2.061	0.788	3.523	
WCP	1.067	1.864	3.388	4.473	0.803	2.626	3.043
WHR	1.207	1.218	1.929	2.313	0.939	2.871	5.234	3.802

*ECP, Eastern Coastal Plains; EHR, Eastern Himalayan Region; EPH, Eastern Plateau and Hills; GPH, Gujarat Plain and Hills; LGP, Lower Gangetic Plains; MGP, Middle Gangetic Plains; UGP, Upper Gangetic Plains; WCP, Western Coastal Plains and Ghats; WHR, Western Himalayan Region.*

### Population Structure of the Indian ORSC Wild Rice Accessions

**FIGURE 5:**
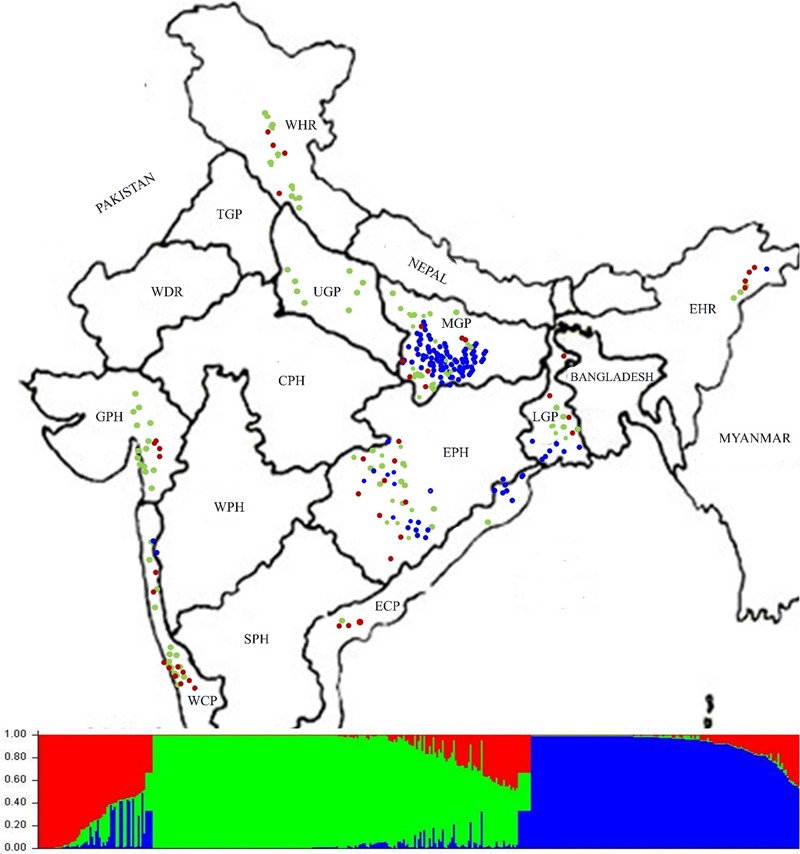
Collection site map of 395 Indian ORSC wild rice accessions (with no missing genotyping data) from different states of India and their population structure based on genome-wide 48-plex GoldenGate SNP assay. Color-coding depicts sub-populations as determined by the STRUCTURE 2.3.4 software (Red: Pro-Indica, Green: Pro-Aus, Blue: Mid-Gangetic). WHR, Western Himalayan Region; EHR, Eastern Himalayan Region; LGP, Lower Gangetic Plains; MGP, Mid Gangetic Plains; UGP, Upper Gangetic Plains; EPH, Eastern Plateau and Hills; ECP, Eastern Coastal Plains; WCP, Western Coastal Plains and Ghats; GPH, Gujarat Plains and Hills; TGP, Trans-Gangetic Plains; WDR, Western Dry Region; CPH, Central Plateau and Hills; WPH, Western Plateau and Hills; SPH, Southern Plateau and Hills.

**FIGURE 6:**
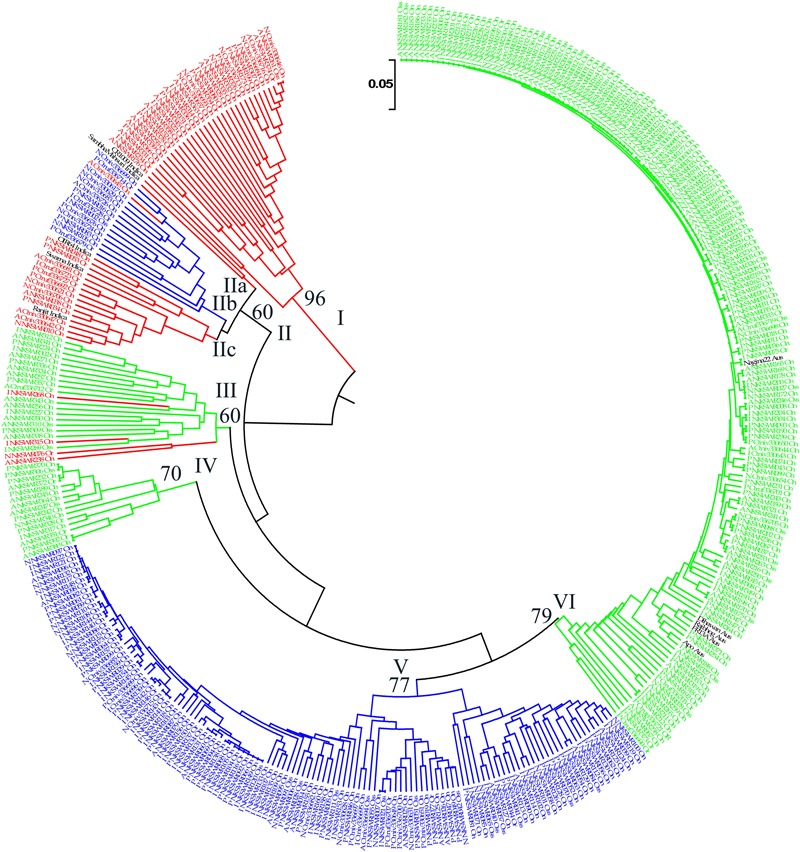
Unweighted pair-group method of arithmetic average based dendrogram of 395 Indian ORSC wild rice accessions and five each of *O. sativa* ‘Aus’ and ‘Indica’ rice cultivars based on 48-plex SNP assay with no missing data. Prefix A, I, P, and N in the accession names represent *pSINE1*-based ecotypes annual, intermediate, perennial, and unknown, respectively; and suffix Or, On, and INT represent morpho-taxonomic classification in to *O. rufipogon, O. nivara*, and *Oryza sativa f. spontanea*, respectively. Sub-populations identified by STRUCTURE 2.3.4 software are color-coded (Red: Pro-Indica, Green: Pro-Aus, Blue: Mid-Gangetic). Bootstrap values are shown at the nodes of the major clustures.

For determining population structure 418 Indian ORSC wild rice accessions and five each of the Aus and Indica rice cultivars were genotyped using genome wide 48-plex SNP array (**Supplementary Table S7**). The Bayesian model based analysis of population structure of 395 accessions using 48-plex SNP with no missing data showed optimum population structure at *K* = 3, suggesting three major sub-populations in these accessions. Sub-population I (colored red in **Figure 5**) comprising 12.66% of the accessions and sub-population II (colored green in **Figure 5**) accounting for 49.88% of the accessions both have predominance of *pSINE1* annual and perennial ecotypes, while population III (colored blue) accounting for 37.46% of the accessions has predominance of *pSINE1* intermediate and perennial ecotypes. The mean Fst values for population I, II, and III were 0.6412, 0.7393, and 0.9345, respectively (**Supplementary Table S8**), suggesting significant proportion of admixture types in the Indian ORSC sub-populations I and II. Based on our earlier study with a subset of these wild rice accessions along with Indica and Aus rice cultivars and co-clustering of known Indica and Aus rice cultivars in the present study, the sub-populations I, and II are designated ‘Pro-Indica,’ ‘Pro-Aus’ populations, respectively (**Figure 6** and **Supplementary Table S8**). Interestingly, the Fst values of the 10 Indica and Aus rice cultivars used in this study show substantial admixture of Mid-Gangetic blood in all of them except Nagina 22. Pro-Indica and Pro-Aus sub-populations were distributed widely throughout India but sub-population III was concentrated mainly in the Mid Gangetic Plains and Eastern Coastal Plains, and was designated Mid-Gangetic population (**Figures 5, 6**). Average distance between individuals in the same cluster (expected heterogeneity) estimated by the STRUCTURE software were significantly different for the sub-populations I, II, and III, with values of 0.1498, 0.1353, and 0.0262, respectively. Admixture types with Fst values of less than 0.9 were observed in each sub-population with the maximum of 54.23% admixture types in sub-population-I, followed by 23.8% in sub-population-II and 22.0% in sub-population-III. This indicates substantial level of natural intercrossing among the three sub-populations of Indian ORSC, particularly with the Pro-Indica sub-population.

It was interesting to note that Upper Gangetic Plains, Western Himalayan Region and Western Coastal Plains and Gujarat Hills and Plains zones have the predominance of Pro-Aus sub-population, with minor presence of Pro-Indica type in the WHR, WCP, and GHP zones. The Upper Gangetic Plains and northern parts of the Mid-Gangetic Plains have exclusive presence of Pro-Aus sub-population (**Figure 5**). Apart from its predominance in the MGP region, the Mid-Gangetic sub-population has significant presence in the Eastern Coastal Plains and Eastern Plains and Hills zones. In contrast to Pro-Aus and Mid-Gangetic sub-populations Pro-Indica sub-population did not dominate any specific agro-climatic zone. In order to examine the specific geographical distribution of pure representatives of the three sub-populations, accessions with Fst values of greater than 0.9 were selected and plotted in the map but there was no significant change in the pattern of geographic distribution of the three sub-populations across India (**Supplementary Figure S3**).

Diversity analysis based on the 48-plex SNP array data grouped 395 wild rice accessions and 10 rice cultivars into six clusters. Color-coding of the accessions according to their population structure showed that clusters I and V represent Pro-Indica and Mid-Gangetic sub-populations, respectively, whereas clusters IV and VI represent Pro-Aus sub-population (**Figure 6**). Clusters II and III were of mixed type where cluster II has individuals from Pro-Indica and Mid-Gangetic sub-populations while cluster III has individuals from Pro-Indica and Pro-Aus, sub-populations. Cluster-IV, although well separated from the major Pro-Aus cluster VI, also has accessions belonging to Pro-Aus sub-population only. Five Aus rice cultivars were nested in the major Pro-Aus cluster VI, but five Indica rice cultivars were grouped with the mixed type cluster II. Though cluster II was further split into three sub-clusters and Indica rice cultivars were nested with Pro-Indica wild rice sub-clusters IIa and IIc (**Figure 6**). Interestingly, accessions belonging to the minor Pro-Aus clusters III and IV came from six diverse Agro-climatic zones of India. However, mixed cluster II came from EPH, ECP, LGP, and MGP zones all in the eastern part of India showing that diversity analysis does group the genotypes into additional clusters according to their geographical origin while population structure analysis reveals their inherent genetic structure based on the ancestral lineage (**Supplementary Table S9**).

Although the optimum number of sub-populations determined by Structure Harvester based on the Δ*K* plot was three, we also examined population structures at different *K*-values in the range of *K*2–*K*9 to see how the Pro-Indica, Pro-Aus, and Mid-Gangetic sub-populations split further into smaller groups and if there was any correspondence between theses smaller groups at higher *K*-values with the Agro-climatic zones (**Supplementary Figure S4** and **Supplementary Table S9**). The analysis revealed that at *K*-values of greater than five large numbers of accessions showed admixture types and there was no clear correspondence between population structure and agro-climatic zones. Up to *K*4 the Pro-Indica and Pro-Aus sub-populations did not split, but the Mid-Gangetic population was split into two subgroups (1 and 3), whereas at *K*5 the Pro-Aus sub-population also split into two subgroups (2 and 3) and Mid-Gangetic population remained split into two subgroups (4 and 5) but with no obvious correlation of the subgroups with agro-climatic zones (**Supplementary Figure S4**). Interestingly, at *K*6 the two subgroups of Mid-Gangetic population once again merged into a single group but other two sub-populations showed further splitting and admixtures. Hence, for further analysis we used the optimum population structure of *K*3.

**FIGURE 7:**
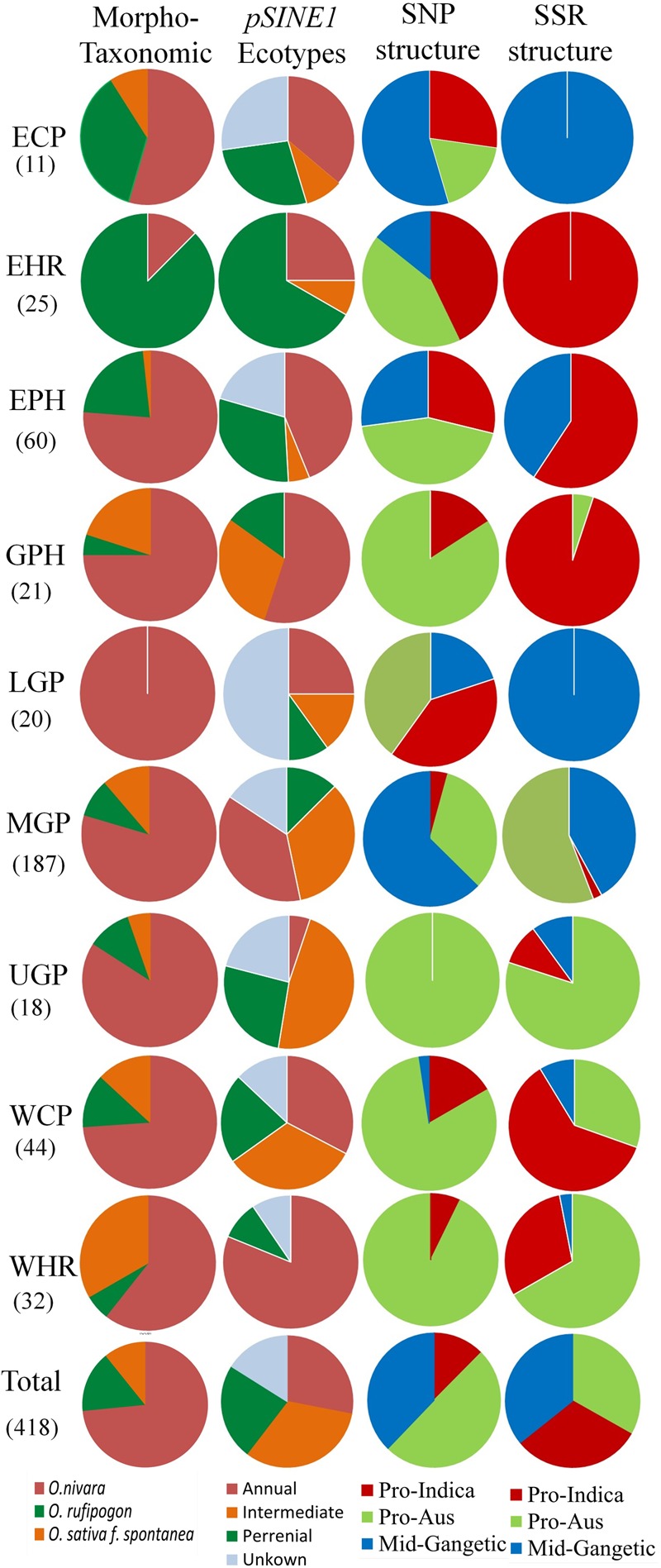
Proportions of different morpho-taxonomic species and *pSINE1* ecotypes of 418 accessions, in comparison to SNP and SSR based population structures of 395 accessions of ORSC wild rice accessions collected from nine different agro-climatic zones of India.

A comparative analysis of the distribution of morpho-taxonomic species groups, *pSINE1* ecotypes and the three population structures obtained with the SNP and SSR markers in different agro-climatic zones showed a broad but not exact agreement among the different classifications (**Figure 7**). According to the morpho-taxonomic classification *O. nivara* was the predominant species in all agro-climatic zones except the EHR zone where *O. rufipogon* was predominant species. *O. rufipogon* was also present though in minor proportion in all the agro-climatic zones except LGP which was occupied entirely by *O. nivara*. The intermediate type *O. sativa f. spontanea* was also present in all the zones except EHR and LGP, and was the second most dominant category in the WHR and GPH zones. The *pSINE1* ecotypes showed a similar distribution pattern to the morpho-taxonomic species groups in different zones, except that the proportion of perennial (*O. rufipogon*) and intermediate ecotypes was almost doubled and that of annual ecotype (*O. nivara*) was halved. The new category of unknown ecotype was also distributed in seven out of the nine zones, and represented fifty percent of the accessions from LGP zone (**Figure 7**).

The agro-climatic zonal distribution of SNP-based population structure showed a broad agreement with SSR-based structure, but with some significant differences. Firstly, the overall proportion of Pro-Aus type accessions was reduced and that of Pro-Indica type was enhanced in the EHR, EPH, GPH, and WCP zones. Also, with SSR-based structure the ECP and LGP zones were fully occupied by the Mid-Gangetic population, whereas with SNP classification these zones showed substantial presence of all the three sub-populations. The interchange of population structure between SNP and SSR markers based classification was largely along the agro-climatic zones (**Figure 7** and **Supplementary Table S9**). However, we have preferred SNP-based population structure because of highly reproducible bi-allelic markers with no missing or ambiguous genotyping scores, and also high number of marker loci in the SNP assay (48 loci) as compared to the HvSSR assay (24 loci). According to SNP assay the Pro-Indica sub-population was present in all the agro-climatic zones except UGP, and it was a predominant group in ECP, EHR, EPH, and LGP zones in the eastern part of India. The Pro-Aus sub-population was present in all the agro-climatic zones without exceptions, and was highly predominant group in UGP, WHR, GPH, and WCP zones in the north-western region of India and the largest group even in the EHR, EPH, and LGP zones in the eastern India. The Mid-Gangetic population was most predominant in the MGP followed by ECP zone, and substantial presence in the geographically adjacent LGP and EPH zones (**Figure 7**).

## Discussion

Altered genetic architecture concomitant with reduced genetic diversity of crop plants has had a profound impact on modern agriculture (Pusadee et al., 2009). Crop domestication and artificial selection (breeding) have genetically structured the cultivated rice varieties. However, unknown evolutionary forces are responsible for shaping the genetic makeup of natural wild rice populations. According to some estimates, modern rice varieties have retained only about 20% of the genetic diversity present in their wild relatives (Londo et al., 2006). This loss of genetic diversity and monoculture has led to the frequent breakdown of resistance to common diseases. Dearth of useful genes in the cultivated rice varieties and landraces has made it obligatory to conserve and utilize the crop wild relatives. India has tremendous diversity of wild rice spread over wide geographical regions, which requires expeditious expeditions to collect and conserve this fast-depleting genetic resource. It was clearly visible during our explorations that the existence of wild rice is under tremendous threat due to clean cultivation, rapid urbanization, and other developmental activities. Hence, there is an urgent need for *ex situ* conservation of representative germplasm and also *in situ* conservation efforts to protect the remaining habitats of ponds and wetlands harboring wild rice diversity. In this study we have covered but a limited number of stress hotspots and habitats in the vast river basins of Ganga and Brahmaputra, wetlands and upland rice growing areas of Bihar, Assam, Himachal Pradesh, Uttar Pradesh, and Uttarakhand, alkaline soil areas of Uttar Pradesh, and coastal regions of Gujarat, Goa, and Odisha to generate a unique set of collection of Indian wild rice that is representative of the genetic diversity of these regions.

Classification of the accessions into respective taxonomic groups is the primary step for utilization of germplasm. Traditionally differential ecological and morphological features have been used for the classification of wild rice accessions to species and subspecies levels (Morishima, 2001). We recorded substantial morphological variation among the different accessions of Indian ORSC wild rice. Based on morphological traits, we identified three taxonomic groups viz. *O. nivara, O. rufipogon*, and *O. sativa f. spontanea.* Morphologically, *O. nivara* Sharma et Shastry has been described as a distinct species from *O. rufipogon* Griff. (Sharma and Shastry, 1965). Banaticla-Hilario et al. (2013) has also reported that Indian *O. rufipogon* and *O. nivara* have diagnostic morphological features such as anther length and spikelet width. But internationally acclaimed ‘The Plant List’ database considers *O. nivara* as a synonym of *O. rufipogon*. Since classification based on morphological traits is less precise, we also used *pSINE1* markers to categorize the accessions into ecotype groups described earlier by Cheng et al. (2002) for the classification of AA genome ORSC accessions into three ecotypes, namely annual, perennial, and intermediate. Based on *pSINE1* markers, we identified a new unknown category of ORSC wild rice not described earlier. The PCA plot generated by morphological descriptors and ecotype-specific *pSINE1* markers showed the ecotypes were dispersed across the PCA axes indicating continuous diversity in wild rice. The dynamic evolutionary process has ensured that the changes under natural selection have occurred in a continuous manner, and natural outcrossing has resulted in some intermediate types between annual (*O. nivara*) and perennial (*O. rufipogon*) and also between cultivated *O. sativa* and wild rice is present among the wild rice populations (Morishima et al., 1961). It was clear from our analysis that *O. nivara* was the predominant morpho-taxonomic species across India.

The SNP and SSR polymorphism have evolved at different rates. While the size of SSR increases due to recombinational slippage and unequal crossing over, SNPs are created by spontaneous mutations resulting in base substitutions. Bi-allelic SNP markers have lesser discrimination between cultivars in comparison to multi-allelic SSR markers on per locus basis, therefore SSR are more suitable for diversity analysis (Coates et al., 2009). However, population structure analysis requires genome wide unlinked markers with high success rate and reproducibility (Pritchard et al., 2000). We found that percentage of admixture types in ORSC accessions was higher with SNP markers as compared to SSR markers. This was consistent with the results of our previous study showing superiority of SNP markers for population structure analysis (Singh N. et al., 2013).

Unlike the cultivated rice, genetic variation in wild rice is free from domestication or other artificial selection processes (Vaughan et al., 2005). Genetic diversity and cluster analysis based on HvSSR markers revealed that the present wild rice collection was highly diverse as no two accessions were identical for the marker loci analyzed. In order to understand the genetic relationship between geographically separated populations, genetic parameters and molecular variance were compared between populations of different ecotypes and agro-climatic zones. The proportion of molecular variation between individuals was much higher than those between populations of different ecotypes or agro-climatic zones, indicating that each population has a mixture of genetically diverse individuals. The overall high genetic diversity of the Indian ORSC wild rice collection was in agreement with the rationale that wild rice offers a much richer source of genetic variation than cultivated rice (Sun et al., 2001; Singh N. et al., 2015). Presence of low level of heterozygosity supported the view that small amount of outcrossing was common in the Indian ORSC wild rice. Further, the clustering pattern based on genome wide HvSSR markers was largely but not entirely in sink with the geographical distribution of the accessions, suggesting no strict reproductive isolation of the three sub-populations. Presence of accessions from MGP zone in all the three major clusters showed that individual accessions of this population shared common alleles with other populations, suggesting that wild rice germplasm may have migrated either naturally or by humans to different agro-climatic regions of India. Isolation by distance analysis found significant but poor correlations between populations from different agro-climatic zones; supporting the view that in a natural population gene flow is inversely related to geographical distance (Slatkin, 1993). Pairwise analysis of migrants per generation based on Fst data matrix also showed comparatively higher level of gene flow between geographically adjacent populations as compared to distant populations. Thus, eco-geographical isolation, outcrossing and gene flow are probably the forces responsible for the genetic structure of the Indian ORSC wild rice populations from different agro-climatic zones (Banaticla-Hilario et al., 2013). In addition, the two sympatric species (*O. nivara* and *O. rufipogon*) have open floral structure and hence they frequently outcross with each other and with cultivated rice (Chen et al., 2004). Considerable level of gene flow between *O. nivara* and *O. rufipogon* has also been reported earlier (Zhou et al., 2008).

Model based population structure analysis using genome wide SNP markers grouped the Indian ORSC wild rice accessions into three sub-populations, supporting the existence of genetically distinct subgroups. This also indicated that the genetic variation among collections from different agro-climatic zones were associated with their ancestry rather than geographic distribution. Fst values of individuals in different sub-populations indicate that a significant proportion of the accessions are admixtures of the three sub-populations (**Figure 5** and **Supplementary Table S8**). Based on the Fst values, Pro-Indica sub-population showed the highest proportion of admixture types, followed by Pro-Aus population, while Mid-Gangetic population has the least proportion of admixture types. Pro-Indica and Pro-Aus sub-populations, both showed pan India distribution but Pro-Aus sub-population was predominant in the UGP, WHR, GPH, and WCP zones of north-western India. Similarly, the Pro-Indica sub-population was present throughout India but was predominant in the EHR and LGP zones of eastern India. Similar overlapping geographical distribution of wild rice sub-populations with closeness to different domesticated cultivar groups have been reported recently by Civáň et al. (2015). In contrast, the accessions of Mid-Gangetic sub-population showing no closeness to major rice cultivar groups and were confined to the Gangetic plains, and represented a conserved wild rice sub-population, although based on Fst values all the selected Indica and Aus rice cultivars, except Nagina 22 showed significant gene flow from the Mid-Gangetic sub-population. However, this may be due to breeding history of these cultivars. Significant outcrossing has been reported for weedy rice (*O. sativa f. spontanea*), which can cross frequently with both cultivated and wild rice and produce admixture types (Pusadee et al., 2013). Thus, two types of admixtures have been reported one between *O. nivara* and *O. rufipogon* (Morishima et al., 1961) and anther one between *O. rufipogon* and *O. sativa* (Rathore et al., 2013). Earlier we have identified two Indian wild rice sub-populations closely related to the Indica and Aus rice cultivars (Singh N. et al., 2015), which was confirmed in the present and the groups were designated Pro-Indica and Pro-Aus wild rice sub-populations described here. The Mid-Gangetic sub-population was not related to any cultivated rice group, however, it may be related to *O. rufipogon* populations rIDN1 and rINDM2 from the same geographical region (Liu et al., 2015). Previous studies have also shown that from two *O. rufipogon* sub-populations, one corresponds to Indica rice cultivars (Huang P.U. et al., 2012). In another study describing three sub-populations of wild rice, one corresponded to Indica, and another to Japonica rice cultivar group (Huang X. et al., 2012). Recently, Kim et al. (2016) identified six sub-populations of global ORSC accessions, where Aus rice cultivar group corresponded to annual sub-population, Japonica to perennial sub-population, Indica to diverse annual and perennial sub-populations, while other three sub-populations were genetically highly divergent groups of wild rice. In all these studies one predominant population of wild rice did not correspond to any of the cultivated rice groups. Hence, among multiple wild rice sub-populations, one is conserved and may assist in identifying the course of rice domestication and serve as source of novel genes for rice improvement.

Realizing the importance of wild rice germplasm, we have screened our collection for resistance to different abiotic and biotic stresses and potential donors have been identified for drought (Singh B.P. et al., 2015) salinity (Mishra et al., 2016a,b) and flooding tolerance; and also resistance to biotic stresses such as rice blast, bacterial leaf blight and sheath blight (our unpublished results). Indian ORSC wild rice is easily crossable to cultivated rice; therefore trait specific introgression lines are easily generated. Detailed information including passport data, morphological features, molecular markers, and results of screening for tolerance to different abiotic stresses are available at our web-enabled database^6^.

## Conclusion

A fresh collection of 418 ORSC wild rice accessions was made from diverse agro-climatic zones of India and classified on the basis of morphological features in to *O. nivara, O. rufipogon* and intermediate *O. sativa f. spontanea* types. The collection was also grouped into four ecotypes, namely annual, perennial, intermediate, and unknown on the basis of *pSINE1* markers. There was predominance of *pSINE1* annual ecotype in *O. nivara* and perennial ecotype in *O. rufipogon*, whereas most of the accessions from spontanea type intermediate morphology group also showed annual ecotype. Majority of the accessions from the new unknown ecotype belonged to *O. nivara* or *O. sativa f*. *spontanea* but rarely to *O. rufipogon*. Analysis of molecular variation established very high proportion of variation between individuals, followed by within individuals (due to heterozygosity) and least proportion of variation was between ecotypes or populations from different agro-climatic zones. Model based population structure analysis using genome wide SNP markers classified the accessions into three sub-populations which show close correspondence with Indica and Aus rice cultivar groups and pure wild rice populations, designated Pro-Indica, Pro-Aus and Mid-Gangetic sub-populations, respectively. There was wide eco-geographical distribution of the Pro-Indica and Pro-Aus sub-populations, while Mid-Gangetic population was limited to the Gangetic plains whereas north-western region of India was occupied predominantly by the Pro-Aus sub-population and the eastern region by the Pro-Indica sub-population. This information will be useful for understanding the process of rice domestication and utilization of wild rice resources for varietal improvement.

## Author Contributions

BS: collection and maintenance of wild rice, phenotyping, genotyping using SSR, SNP and *p-SINE1* markers, data analysis and manuscript writing; NS: designing of SNP genotyping panel; SM: morphological trait measurements, SNP genotyping and data analysis; BPS: collection of wild rice, SSR genotyping; KT: design and maintenance of database; VR: manuscript editing; AS: guiding field experiments; NKS: wild rice collection, planning and supervision of the experiments, manuscript editing and finalization.

## Conflict of Interest Statement

The authors declare that the research was conducted in the absence of any commercial or financial relationships that could be construed as a potential conflict of interest.

## Footnotes

1 http://www.theplantlist.org

2 http://irri.org/our-work/research/genetic-diversity/international-rice-genebank

3 http://www.nbpgr.ernet.in:8080/PGRPortal/

4 http://nksingh.nationalprof.in:8080/iwrdb

5 http://snp-seek.irri.org/

6 http://nksingh.nationalprof.in:8080/iwrdb

## Supplementary Material

FIGURE S1 Physical map positions of SNPs in the genome wide 48-plex Illumina GoldenGate assay designed and used for determination of population structure of Indian wild rice accessions.

FIGURE S2 Principal component analysis (PCA) of morphological traits and *pSINE1* ecotypes among 418 Indian ORSC wild rice accessions. PCA explains 13.62% (*x*-axis) and 10.14% (*y*-axis) of total variations. Symbols identify germplasm groups based on ecotype-specific *pSINE1* markers (A, annual; I, intermediate; P, perennial; U, unknown).

FIGURE S3 Collection site map of 260 ORSC Indian wild rice accessions with Fst values of >0.9 for sub-population structures. Color-coding depicts sub-population of the accessions as determined by the STRUCTURE 2.3.4 software. (Red: Pro-Indica; Green: Pro-Aus; Blue: Mid-Gangetic populations). Google Earthv7.1.4.1529, US Department of State Geographer Data SIO, NOAA, US Navy, NGA, GEBCO ©2017 image Landset/Copernicus.

FIGURE S4 Comparative population structures of 395 Indian ORSC wild rice accessions at different *K*-values (*K*2–*K*9) using STRUCTURE 2.3.4 software based on genome-wide 48-plex SNP assay.

TABLE S1 Geographical location and GPS informations of 418 *O. rufipogon* Griff. species complex (ORSC) wild rice accessions collected from different agro-climatic zones of India.

TABLE S2 **(A)** Data on 46 morphological descriptors evaluated on 418 Indian *O. rufipogon* Griff. species complex (ORSC) wild rice accessions. **(B)** ANOVA of nine quantitative traits showing *F*- and *P*-values for the level of significance.

TABLE S3 Pseudomolecule physical positions, gene Id, flanking nucleotide sequence, output quality scores and Illumina assay Id of SNP markers designed at one design ability rank for model based population structure analysis using 48-plex Illumina GoldenGate assay.

TABLE S4 Genotyping scores of nine *pSINE1*-based markers used for species and ecotype classification of 418 Indian *O. rufipogon* Griff. species complex (ORSC) wild rice accessions.

TABLE S5 Genotyping scores of 24 HvSSR markers used for analyzing molecular variance and genetic diversity of 418 *O. rufipogon* Griff. species complex (ORSC) wild rice accessions collected from different agro-climatic zones of India.

TABLE S6 AMOVA of four *pSINE1* ecotypes (A, annual; I, intermediate; P, perennial; U, unknown) of Indian ORSC wild rice accessions based on SSR markers.

TABLE S7 Genotyping scores of 48-plex genome wide SNP markers used for defining the population structure of 395 wild rice accessions collected from different agro-climatic zones of India.

TABLE S8 Fst values of 395 Indian *O. rufipogon* Griff. species complex (ORSC) wild rice accessions and 5 each of Indica and Aus rice cultivars classified in to three sub-populations by model-based population structure analysis using genome wide 48-plex GoldenGate SNP assay.

TABLE S9 Correspondence between HvSSR and SNP markers based population structures at *K*3, SNP dendrogram clustering and comparative analysis of population structures at different *K*-value (*K*2–*K*9) with agro-climatic zones of Indian ORSC wild rice accessions. Accessions showing mismatch between SSR and SNP based-population structures are shaded in different colors.
