# Supplementary figures and images for: Morphological and Molecular Data Reveal Three Distinct Populations of Indian Wild Rice Oryza rufipogon Griff. Species Complex
Source: Front Plant Sci. 2018 Feb 7;9:123. doi: 10.3389/fpls.2018.00123 (PMC5808308; doi:10.3389/fpls.2018.00123)

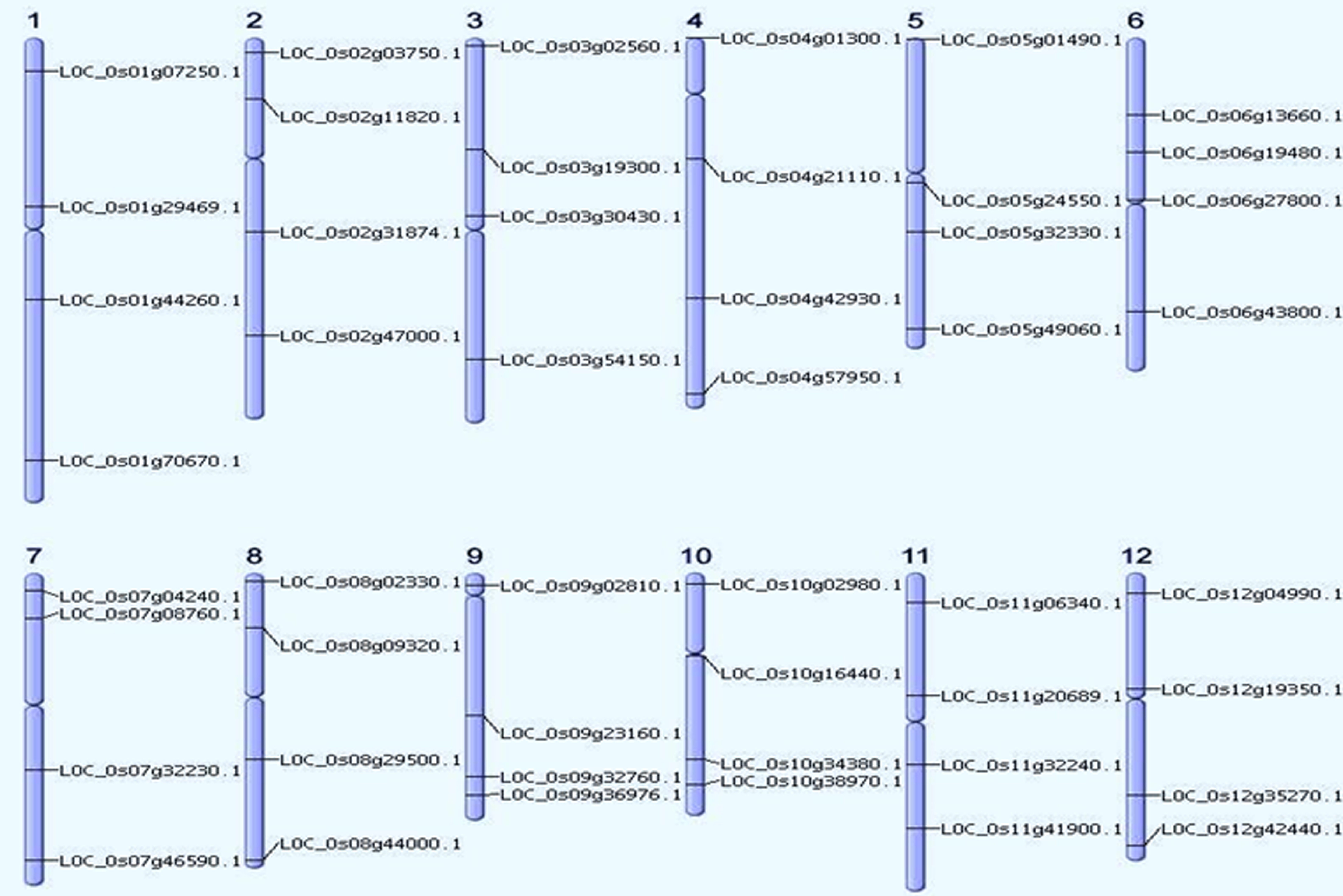

Supplement: FIGURE S1 — Physical map positions of SNPs in the genome wide 48-plex Illumina GoldenGate assay designed and used for determination of population structure of Indian wild rice accessions. [file Image_1.JPEG]

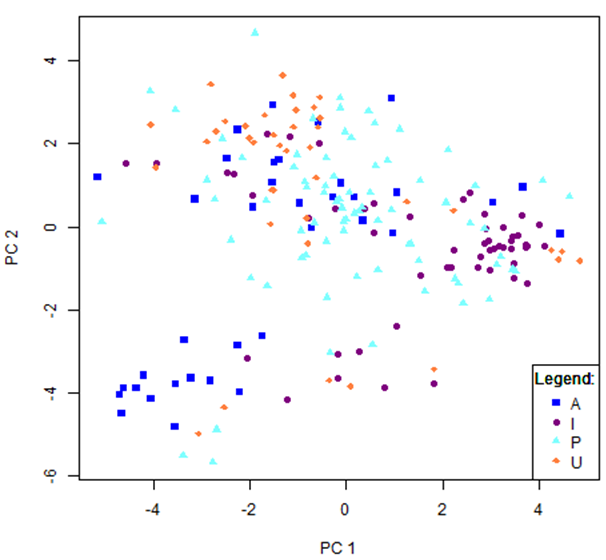

Supplement: FIGURE S2 — Principal component analysis (PCA) of morphological traits and pSINE1 ecotypes among 418 Indian ORSC wild rice accessions. PCA explains 13.62% (x-axis) and 10.14% (y-axis) of total variations. Symbols identify germplasm groups based on ecotype-specific pSINE1 markers (A, annual; I, intermediate; P, perennial; U, unknown). [file Image_2.TIF]

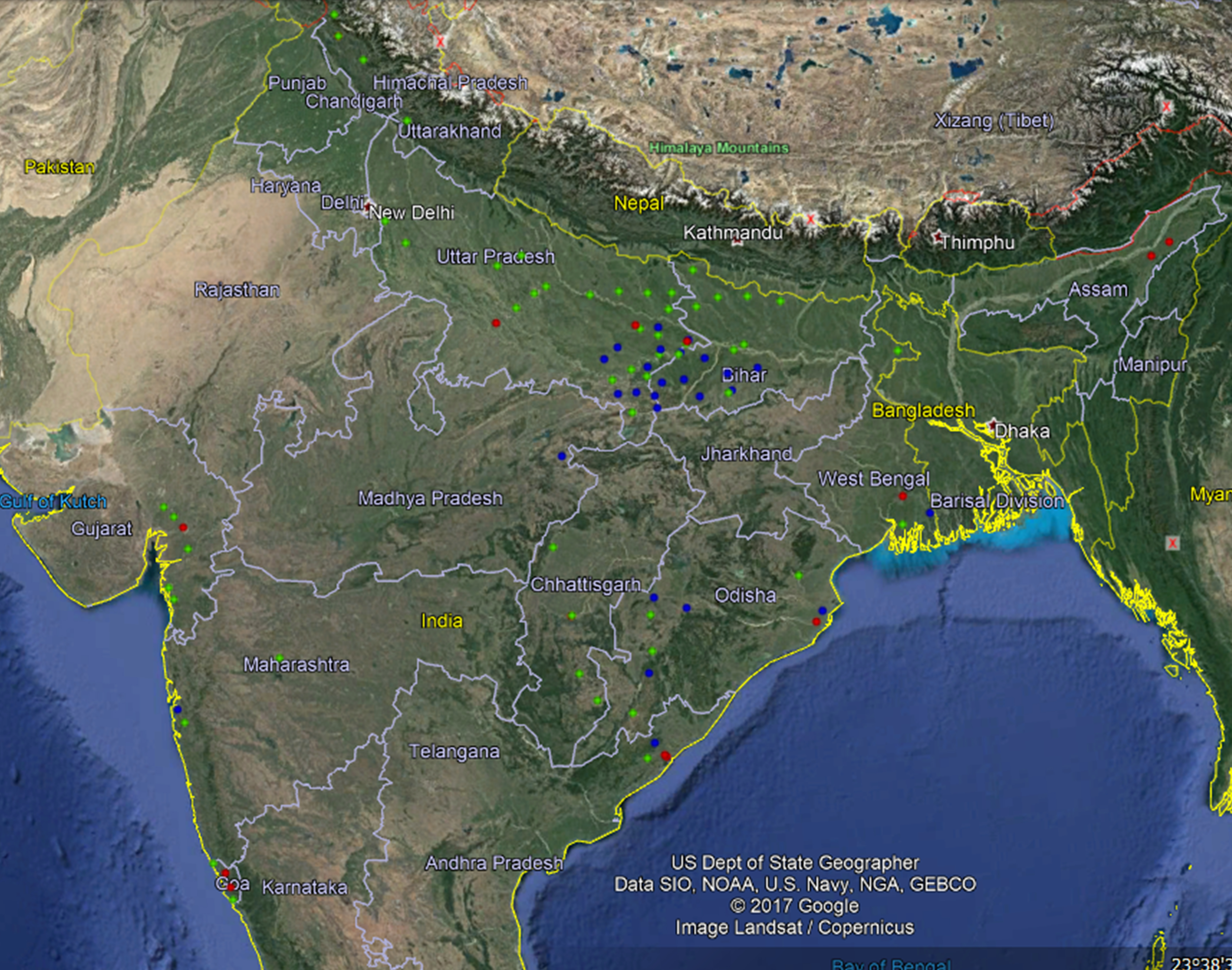

Supplement: FIGURE S3 — Collection site map of 260 ORSC Indian wild rice accessions with Fst values of >0.9 for sub-population structures. Color-coding depicts sub-population of the accessions as determined by the STRUCTURE 2.3.4 software. (Red: Pro-Indica; Green: Pro-Aus; Blue: Mid-Gangetic populations). Google Earthv7.1.4.1529, US Department of State Geographer Data SIO, NOAA, US Navy, NGA, GEBCO ©2017 image Landset/Copernicus. [file Image_3.JPEG]

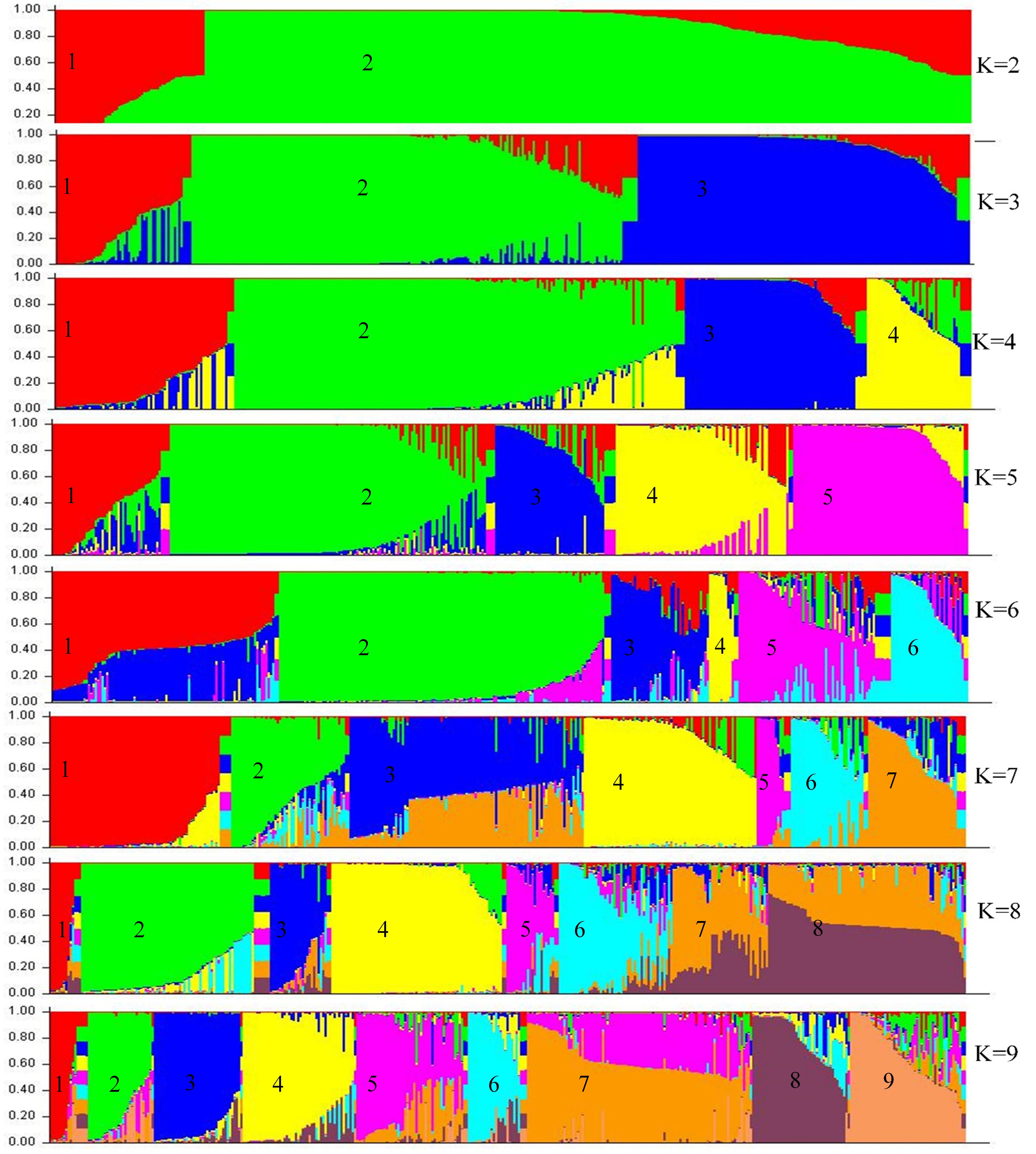

Supplement: FIGURE S4 — Comparative population structures of 395 Indian ORSC wild rice accessions at different K-values (K2–K9) using STRUCTURE 2.3.4 software based on genome-wide 48-plex SNP assay. [file Image_4.JPEG]
